# Different Phenotypes of the Two Chinese Probands with the Same c.889G>A (p.C162Y) Mutation in *COCH* Gene Verify Different Mechanisms Underlying Autosomal Dominant Nonsyndromic Deafness 9

**DOI:** 10.1371/journal.pone.0170011

**Published:** 2017-01-18

**Authors:** Qi Wang, Peipei Fei, Hongbo Gu, Yanmei Zhang, Xiaomei Ke, Yuhe Liu

**Affiliations:** Department of Otolaryngology, Head and Neck Surgery, Peking University First Hospital, Beijing, China; NIDCR/NIH, UNITED STATES

## Abstract

**Objectives:**

By analyzing the different phenotypes of two Chinese DFNA9 families with the same mutation located in the intervening region between the LCCL and vWFA domains of cochlin and testing the functional changes in the mutant cochlin, we investigated the different pathogeneses for mutations in LCCL and vWFA domains.

**Methods:**

Targeted next-generation sequencing for deafness-related genes was used to identify the mutation in the proband in family #208. The probands of family #208 and family #32 with the same p.C162Y mutation were followed for more than 3 years to evaluate the progression of hearing loss and vestibular dysfunction using pure-tone audiometry, caloric testing, electrocochleogram, vestibular-evoked myogenic potential, and video head-impulse test. The disruption of normal cleavage to produce secreted LCCL domain fragments and the tendency to form aggregations of mutant cochlins were tested by *in vitro* cell experiments.

**Results:**

The two families showed different clinical symptoms. Family #32 was identified as having early-onset, progressive sensorineural hearing loss, similar to the symptoms in DFNA9 patients with cochlin mutations in the vWFA domain. The proband of family #208 endured late-onset recurrent paroxysmal vertigo attacks and progressively deteriorating hearing, similar to symptoms in those with cochlin mutations in the LCCL domain. We therefore suggest that the disrupted cleavage of the LCCL domain fragment is likely to cause vestibular dysfunction, and aggregation of mutant cochlin caused by mutations in the vWFA domain is responsible for early-onset hearing loss. The p.C162Y mutation causes either disruption of LCCL domain fragment cleavage or aggregation of mutant cochlin, resulting in the different phenotypes in the two families.

**Conclusion:**

This study demonstrates that DFNA9 families with the same genotype may have significantly different phenotypes. The mutation site in cochlin is related to the pathological mechanism underlying the different phenotypes.

## Introduction

Cochlin is an abundant extracellular matrix protein in the cochlea and vestibule of the inner ear. The full length cochlin contains 550 amino acids, consisting of N-terminal secretary signal peptide, LCCL domain (Limulus factor C, Cochlin, and late gestation protein Lgl1), ivd1, vWFA1 domain (von Willebrand factor A), ivd2 and vWFA2 domain [1, 2]. Cochlin is cleaved *in vivo* in the ivd1 region to form two shorter fragments [3]. Currently, 20 missense mutations and two in-frame deletions have been reported in *COCH* gene that encodes cochlin, including 15 mutations located in exon 4 or 5 encoding the LCCL domain [4–13], and six mutations located in exon 11 or 12 encoding the vWFA domain [14–16]. Only the c.889G>A (p.C162Y) variant was located in exon 7 encoding the ivd1 domain [17]. These missense mutations and in-frame deletions in *COCH* gene are etiologically linked to autosomal dominant nonsyndromic deafness 9 (DFNA9), a disorder characterized by late onset and progressive hearing loss and vestibular dysfunction [18–21].

The phenotype of DFNA9 patients due to mutations in *COCH* gene varies and correlates with the location of the mutation in cochlin [22, 23]. Families carrying mutations in the LCCL domain are more likely to have hearing loss and self-reported vestibular dysfunction, whereas those carrying mutations in the vWFA domain present with severe hearing loss without complaints of vestibular symptoms [20]. Up to the present time, all affected DFNA9 family members eventually endure bilateral moderate-severe to profound hearing loss starting from the high frequencies and slowly progressing to all frequencies with the onset age ranging from the 2nd decade to the 6th decade [15, 24]. Vestibular symptoms including instability, vertigo, dizziness, tinnitus, and fall tendency are reported in those carrying mutations in the LCCL domain. Some patients endure years of hearing loss before encountering vestibular symptoms, while others endure both hearing loss and vestibular symptoms simultaneously [25]. The varied phenotype in patients with the same genotype has also been noted in previously reported DFNA9 families, and the commonly described variations include age of onset and rate of progression of hearing loss.

Recently, we identified a Chinese DFNA9 family (#208) carrying a c.889G*>*A (p.C162Y) mutation located in exon 7 encoding ivd1 in *COCH* gene, the same mutation we previously reported in a DFNA9 family (#32) [17]. The proband in family #208 presented with symptoms of paroxysmal vertigo and hearing loss like Ménière’s disease, while the patients in family #32 had sensorineural hearing loss beginning at around 17 years of age and progressively deteriorating from the high frequencies. In this study, we analyzed the course of the disease in the two probands from the two families. Using *in vitro* experiments, we also investigated the mechanism underlying the pathogenesis due to the p.C162Y mutation. The relationship between genotype and pathological mechanism was then explored in association with information on DFNA9 families previously reported by others.

## Materials and Methods

### Patients

Medical history and pedigree investigation were taken from all members in family #208 in 2012. Audiological tests and vestibular assessment were performed in 2012 and repeated in 2015 for the proband in family #208. The clinical information for the proband in family #32 was collected in 1998 and was used for comparison of phenotype between the two families. Written informed consent was obtained from all participants. This study was approved by the Medical Ethics Committee of Peking University First Hospital.

### Targeted next-generation sequencing

A blood sample was collected from the proband in family #208. Genomic DNA was extracted using the Qiagen blood DNA extraction kit (Qiagen, Hilden, Germany). The targeted exome sequencing included 136 nuclear genes and mitochondrial genes known to be responsible for nonsyndromic or syndromic hearing loss. After the construction of an enrichment library using the MyGenostics GenCap enrichment kit (MyGenostics, Baltimore, MD, USA) [26], the sample was sequenced on an Illumina HiSeq 2000 sequencer (Illumina, San Diego, CA, USA). The SOAPaligner program was then used to align the read sequences to the human reference genome (hg19). After duplicates had been removed using Picard software, the SNPs and InDels were identified using SOAPsnp and GATK programs. Subsequently, the reads were realigned to the reference genome using BWA. The identified SNPs and InDels were annotated using the Exome-assistant program. MagicViewer was used to view the short read alignments and validate the candidate SNPs and InDels. The variants detected by targeted next-generation sequencing were confirmed by Sanger sequencing. The forward primer 5’-CAAGATCTCCATTTGGGAAGG and reverse primer 5’-ACCCACTTGGCCTCCAAAG were used for amplification of exon 7 of *COCH* gene in the family members of this pedigree. PCR products were directly sequenced using an ABI 3730 sequencer.

### Audiological tests

Pure-tone audiometry (PTA) for sensorineural hearing loss diagnosis was performed by standard audiometry (Otometrics, Taastrup, Denmark) in a sound-proofed room according to clinical standards. Tympanometry was performed using a Madsen Otoflex 100 immittance system. The transient evoked otoacoustic emissions (TEOAEs) and distortion product otoacoustic emission (DPOAE) recordings were conducted using a Madsen Capella OAE System.

### Videonystagmography

Eye movements were recorded via the ICS Medical VG-30 infrared camera goggles and subsequent eye movements were analyzed using ICS Medical CHARTR ENG version 4.1 software. Spontaneous nystagmus testing was performed, while Random Saccade and Smooth Pursuit Eye movements were recorded as per nystagmography. Monitored eye movements were analyzed by computer and compared to age-matched and in-house validated normative data in terms of gain, latencies and accuracy of eye movements.

### Caloric testing

This was performed with the patient supine and head anteroflexed at 30°. Irrigation with hot (50°C) and cold (24°C) water was maintained for 30 seconds with a between-stimuli interval of 5 minutes. Symmetry of peak angular slow-phase velocity was analyzed and unilateral canal paresis (UCP) was assumed at a side difference >15%.

### Electrocochleogram (ECochG)

This was performed using SmartEP 3.86 equipment from Intelligent Hearing Systems (Otometrics, Taastrup, Denmark). The exam was conducted using a tympanic electrode (TM–Wick Electrode; ref.: 60173 Otometrics, FL, USA). The electrode was positioned close to the posteroinferior quadrant of the tympanic membrane. The reference electrode was positioned on the mastoid bone of the contralateral ear with the ground electrode on the forehead. Click stimuli and tone bursts at frequencies of 1000 Hz were utilized. A reproducible SP/AP (summating/action potential) ratio of greater than 37% was considered abnormal for the click stimulus.

### Vestibular-evoked myogenic potential (VEMP)

This was recorded from the contracted cervical muscles (cVEMP) via the sternocleidomastoid muscle through stimulation by short-tone bursts (STB, 500 Hz; 95 dBnHL normal hearing level; rise/fall time, 1 ms; plateau time, 2 ms). Surface EMG activities were recorded from Ag/AgCl surface electrodes ipsilaterally placed over the middle part of the muscle. The reference electrode was placed on the upper sternum and the ground electrode was positioned between the eyebrows. Using the ICS ChartrEP 200 (Otometrics, Taastrup, Denmark) and an insertphone type headphone, the recorded VEMPs were measured by the mean latencies of peaks p13 and n23 and the amplitudes of peak p13 to peak n23.

### Video head-impulse test

ICS Impulse (Otometrics, Taastrup, Denmark) was used in this test which recorded the corrective saccades when rapidly turning the head to the side of vestibular weakness. Approximately 20 horizontal head impulses were manually applied to each side with random timing and direction. The same eye was recorded simultaneously with video-oculography. Two data sets were obtained for each recording session to show the reliability of the calculated gains. Video images were analyzed online to calculate eye position and velocity using a pupil detection method. The criterion for a normal vestibular ocular reflex (VOR) velocity gain was that it should be 0.68 or greater, based on head impulse test (HIT) data from 12 previously published healthy asymptomatic subjects.

### Transient expression in HEK293 cells

The mammalian expression plasmid containing the open reading frame of mouse COCH cDNA (NM_001135058) with a DDK-myc tag at the 3’ end was obtained from Origene (Rockville, MD, USA). We also used a site-directed mutagenesis kit (Transgen, Beijing, China) to produce the c.889G*>*A (p.C162Y) mutation in COCH cDNA in the plasmid. The sequences in the wild-type and mutant COCH plasmids were confirmed by sequencing. Human kidney cell line HEK29 cells were divided into two groups: one was transfected with wild-type COCH plasmid, and the other was transfected with the mutant COCH plasmid.

### Mass spectrometry

After 48 hours, the cultured cells were collected in the lysis buffer (50 mM Tris–HCl, pH 7.4, 150 mM NaCl, 1 mM EDTA, 1% Triton X-100) supplemented with protease inhibitor cocktail (M221, AMRESCO) to produce cell lysate. After separating the lysate in SDS–PAGE, the gels were silver-stained and digested for LC-MS/MS analysis. The sample was separated by a 120-min gradient elution at a flow rate of 0.300 μL/min with the Dionex 3000 nano-HPLC system. The Q Exactive mass spectrometer was operated in the data-dependent acquisition mode using Xcalibur 2.1.3 software and a single full-scan mass spectrum was collected with an Orbitrap mass spectrometer (400–1800 m/z, 70,000 resolution) followed by 20 data-dependent MS/MS scans. The MS/MS spectra were searched against the Uniprot database (http://www.uniprot.org/).

### Western blot

The SDS–PAGE gel was transferred onto a nitrocellulose membrane, and the membrane was blotted with 1:1000 rat anti-COCH antibody (MABF267, Millipore) diluted in 5% non-fat milk/Tris-based saline/0.05% Tween 20 solution. The blotted antibody was recognized by 1:10,000 peroxidase-conjugated mouse anti-rat IgG antibody, and developed with the enhanced chemiluminescence method (ECL; Immobilon Western Chemiluminescent HRP Substrate, Millipore).

### Immunofluorescence

The transfected cells were fixed in 4% paraformaldehyde. After washing three times, they were incubated in a blocking solution (10% goat serum/PBS/0.25% Triton X-100) for 1 h. The cells were then incubated with 1:400 mouse anti-FLAG antibody (# A02010, Abbkine) in the blocking solution at 4°C overnight. After washing, the cells were incubated with 1:500 Alexa Fluor 488-conjugated goat anti-mouse IgG (A11034, Molecular Probes) in PBS at room temperature for 1 h. After washing three times and staining with DAPI, the slides were observed with a confocal microscope.

## Results

### Missense mutation in *COCH* gene in family #208 and family #32

**Fig 1:**
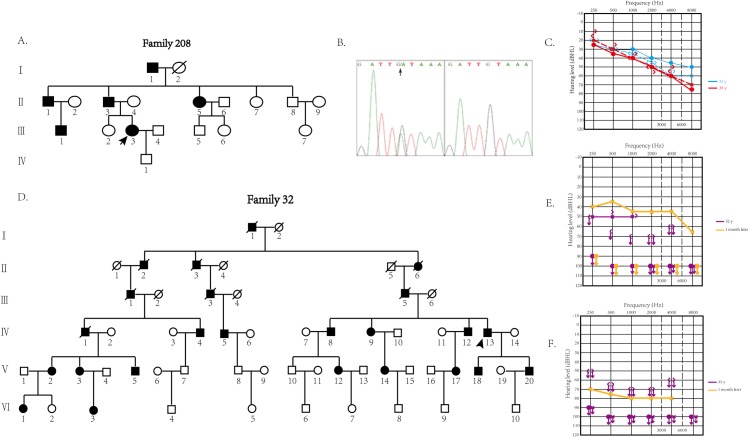
**A. The pedigree of family #208.** Open symbols, unaffected; solid symbols, affected. Squares, male; circles, female; slashed, deceased individual. Slanting arrow, the proband. **B. The heterozygous c.889G>A, p.C162Y variant.** This variant is located in the seventh exon of *COCH* gene and was identified in the affected members in family #208. **C. Results of pure tone audiometry for two visits of proband in family #208 with hearing loss.** Threshold data were obtained from both ears. Blue symbols, results from first visit; red symbols, results from the follow-up visit. The results presented bilateral sensorineural hearing loss with a decreasing pattern, involving the low frequencies. The threshold of the patient’s audiogram showed a mild decrease in the high frequencies in the follow-up visit. **D. The pedigree of family #32.** Open symbols, unaffected; solid symbols, affected. Squares, male; circles, female; slashed, deceased individual. Slanting arrow, the proband. **E. Results of the pure tone audiometry of proband in family #32 at the first visit.** Severe sensorineural hearing loss in the left ear (50 dB HL by 6-tone average) and complete deafness at all frequencies in the right ear were shown (purple symbols). After 1 week, reexamination of the audiogram showed a 10 dB recovery in low frequencies (yellow symbols). **F. Results of the pure tone audiometry of the proband in family #32 at the second visit.** Two years later, the proband suffered another episode of sudden deafness at all frequencies (purple symbols), but the treatment showed limited effects according to the results 1 week later (yellow symbols).

Family #208 is a Chinese family with DFNA9 of autosomal dominant inheritance spanning four generations (Fig 1A). Six family members ranging from 35 to 80 years of age were diagnosed as having sensorineural hearing impairment by pure-tone audiometry. Some patients also had symptoms of tinnitus and dizziness. The proband was characterized by recurrent vertigo and progressive deterioration of hearing loss.

A blood sample from the proband in family #208 was subjected to targeted next-generation sequencing, which included 136 nuclear genes and the mitochondrial genes known to be responsible for nonsyndromic or syndromic hearing loss (S1 Table). Sequencing data were analyzed using publicly available databases such as dbSNP 135, HapMap database, 1000 Genomes Project and a local control database, and then confirmed by PCR-Sanger sequencing. Seven candidate variants were identified in the proband (S2 Table). Based on the characteristics of nonsyndromic hearing loss and autosomal dominant inheritance in this family, three variants causing recessive hearing loss were excluded. We then used SIFT and PolyPhen-2 websites to predict functional changes of the remaining four variants, and only the c.889G>A (p.C162Y) variant in *COCH* gene (Fig 1B) was predicted to be pathogenic. The proband was heterozygous of the variant by Sanger sequencing, identical to the proband in the Chinese family #32 we reported previously (Fig 1D).

### Clinical characteristics of the probands in family #208 and family #32

The proband in family #208 (pedigree III-3) initially presented with bilateral sensorineural hearing loss of a decreasing pattern involving low frequencies (Fig 1C) associated with tinnitus, aural fullness and self-reported vertigo in her 4th decade of age. The vertigo was recurrent and with head-motion-unrelated spinning. She endured three severe attacks in the first year. During the severe attacks, physical movement was restricted and tinnitus and aural fullness became aggravated without a change in audile ability, which continued for more than 5 hours but not more than 24 hours. Mild attacks often occurred between severe attacks, during which physical activities could continue. In the follow-up period of 3.6 years, the audiogram threshold showed a mild decrease at high frequencies (Fig 1C), severe vertigo attacks and symptoms such as tinnitus and aural fullness persisted. The acoustic immittance testing suggested a normal middle ear. The otoacoustic emission (OAE) tests consistently showed no signal for TEOAE and partially induced DPOAE signals, indicating damage to the cochlea.

In our hearing loss family #32 with the same mutation of c.889G>A (p.C162Y) in *COCH* gene, the affected members presented with early-onset hearing loss in the 2nd decade of life [17]. The proband in family #32 demonstrated severe sudden deafness several times without vertigo. In the first episode, he suffered complete deafness in his right ear. After 3 years, he suffered a sudden deafness attack in the other ear. Pure tone audiometry (Fig 1E) revealed severe sensorineural hearing loss of the left ear (50 dB HL from six-tone average) and right ear (complete deafness at all frequencies). His external auditory canals and tympanic membranes were normal. After treatment with prednisone and ginaton (ginkgo biloba leaf preparation) for 1 month, the audiogram showed a 10 dB recovery at low frequencies. Two years later, the patient suffered another attack of sudden deafness at all frequencies, with limited response to treatment (Fig 1F).

### Vestibular function assessment of the proband in family #208

**Fig 2:**
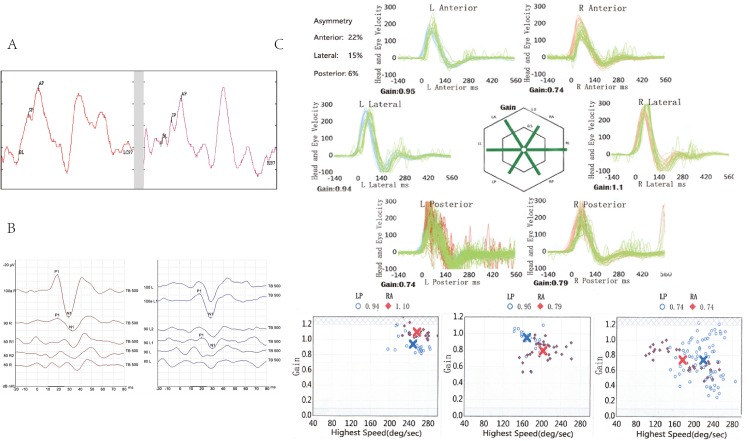
**A. Electrocochleogram results for the proband of family #208 at the follow-up visit.** The ratios of SP to AP were 62% on the left (left ear) and 50% on the right (right ear). **B. VEMP results for the proband of family #208 at the follow-up visit.** The results showed a lower level of amplitude between p13 and n23 in the left ear (right panel). **C. Video head-impulse test results for the proband of family #208 at the follow-up visit.** The VOR velocity gain was greater than 0.68 in all directions. There were no significant abnormalities in the test results.

Normal videonystagmography in the neurotologic examination excluded the central causes of her vertigo. Caloric testing showed unilateral canal paresis on the left side with a side difference of 30% and the abnormalities returned to normal in the follow-up study, consistent with the self-reported degree of vertigo. For the electrocochleogram, the SP/AP ratios were 51% and 38% for left and right ears, respectively, and increased to 62% and 50%, respectively, on follow-up study (Fig 2A), indicating aggravation of the abnormality. The VEMP neuroelectrophysiological assessment technique evaluates otolithic organs of the utricle and saccule. The proband displayed normal latency of p13 and n23 on both sides, and lower amplitude between p13 and n23 on the left side, indicating a weakened function of the utricle on the left side (Fig 2B), consistent with the weakened equilibrium function of the proband. In addition, there were no significant abnormities in video head-impulse test during the interval of vertigo attacks (Fig 2C). The clinical differences between the probands in family #208 and family #32 are summarized in Table 1.

**Table 1:** Clinical characteristics of the probands in family #32 and family #208.

	Onset age	Hearing symptoms	Vestibular symptoms	Assessment of vestibular function				
				VNG	caloric	EcochG	VEMP	HIT
**Proband of Family #32**	2nd decade	+	-	N	N	N	N	N
**Proband of Family #208**^**a**^	4th decade	+	+	-	+	+	+	N
**Proband of Family #208**^**b**^	4th decade	+	-	-	-	+	+	-

+, present

–, not present

N, not available

^a^, during the attack

^b^, during interval of the attacks.

VNG, videonystagmography; caloric, caloric testing; EcodchG, electrocochleogram; VEMP, vestibular-evoked myogenic potential; HIT, video head-impulse test

### Functional analyses of the p.C162Y mutation in cochlin

Mutations in cochlin are gain-of-function mutations resulting in DFNA9. Mutant cochlins may have either abnormal formation of an intramolecular disulfide bond or impaired post-translational cleavage to produce two shorter fragments [21–26]. We tested the functional changes of the p.C162Y mutant cochlin. Basic physical and chemical properties were predicted for wild-type and mutant cochlin. The extinction coefficient was 0.798 for mutant cochlin and 0.773 for wild-type cochlin. The grand average of hydropathicity (GRAVY) was -0.025 for wild-type cochlin and -0.032 for mutant cochlin, suggesting that the mutant cochlin is likely to be insoluble. The most hydrophobic region is located near the 162nd amino acid residue in the mutant cochlin.

**Fig 3:**
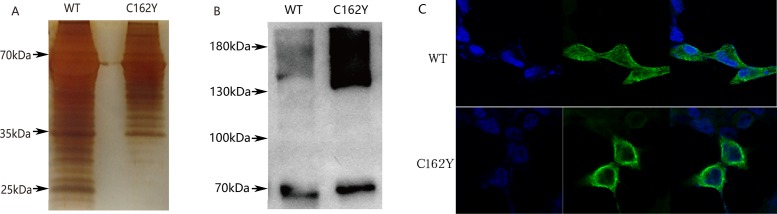
**A. Silver-stained SDS–polyacrylamide gels.** The protein bands located near 25 kDa in the wild-type cochlin lane were obvious compared with the mutant type cochlin lane. This area was subjected to MS analysis. **B. Western blot results.** The results showed that the mutant type cochlin showed an accumulating pattern in the region representing the dipolymers and aggregations (around 140 kDa). **C. Immunofluorescence staining** The fixed HEK293 cells transiently transfected with wild-type or mutant cochlins were prepared for immunofluorescence staining using anti-FLAG antibody and stained with Alexa Fluor 488. The cells transfected with mutant cDNA showed an aggregated pattern around nuclei.

HEK293 cells were transfected with wild-type or c.889G>A (p.C162Y) mutant cochlin cDNA to examine the functional changes of the mutant cochlin. After transfection for 48 hours, the 10x concentrated cultured medium was subjected to SDS-PAGE and silver staining (Fig 3A). The bands near 25 kDa were much weaker in the cultured medium from the cells transfected with mutant cDNA. The most clearly stained band near 25 kDa was cut for mass spectrometry, and the result showed that cochlin was the most abundant protein in this band. Therefore, p.C162Y mutation affected the secretion of shorter cochlin fragments into the medium.

The cell lysate of transfected cells was harvested and separated in non-denatured polyacrylamide gel to conduct western blot using rat anti-cochlin antibody as the primary antibody (Fig 3B). More dimeric cochlin (~130 kDa) and aggregated cochlin (>130 kDa) were found in the cells transfected with the mutant cDNA. The transfected cells were also stained by immunofluorescence to show transfected cochlin and observed under a confocal microscope. The cells transfected with mutant cDNA showed an aggregated pattern around nuclei (Fig 3C).

### Relationship between the mutation location in cochlin and the phenotype of patients

Cochlin mutations and clinical findings in this study and in previous reports are summarized in Table 2. Mutations in the LCCL domain frequently cause the impairment of normal cleavage to produce LCCL domain fragments and significant vestibular symptoms. Mutations in the vWFA domain are less likely to cause severe vestibular dysfunctions as this domain is relatively far from the cleavage site. These data also suggest that mutations in the vWFA domain are more likely to form aggregates instead of dimers because this domain may be involved in the formation of extracellular matrix. Significant aggregation of cochlin is more likely to cause cochlear dysfunctions such as early-onset hearing loss and sudden deafness. The p.C162Y mutation is located between the LCCL and vWFA domains, and is therefore predicted to cause dysfunctions in the cleavage of LCCL domain fragments and the formation of extracellular matrix. The two families we described have the same mutation but different phenotypes so are in accordance with the prediction, yet the pathological processes remain to be clarified.

**Table 2:** Relationship between mutation location in cochlin and abnormal findings.

Origin	Mutation in domain	Amino acid change	Cleavage of LCCL domain	Vestibular symptoms	Cochlin dimer or aggregates	Early-onset hearing loss
de Kok et al. (1999, Netherlands)	LCCL	P51S	Disrupted	+	Dimer	-
Robertson et al. (1998, United States)	LCCL	V66G	Disrupted	+/-	Dimer	-
Robertson et al. (1998, United States and Netherlands)	LCCL	G88E	Disrupted	+	Dimer	-
Pauw et al. (2007, Netherlands)	LCCL	I109T	Disrupted	+	Dimer	-
Robertson et al. (1998, Korea)	LCCL	W117R	Disrupted	+/-	Dimer	-
Hildebrand et al. (2010, United States)	LCCL	F121S	N	+	Dimer	-
Jung et al. (2015, Korea)	LCCL	V123E	Disrupted	-	Dimer	-
Gao et al. (2012, China)	ivd1	C162Y	Disrupted	-	Aggregates	+
This study (China)	ivd1	C162Y	Disrupted	+	Aggregates	_
Cho et al. (2012, Korea)	vWFA2	F527C	N	-	Aggregates	+
Yuan et al. (2008, China)	vWFA2	C542Y	N	-	Aggregates	+

+, presented in the family

-, not presented in the family

N, not identified.

## Discussion

In this study, the probands in the two families with the same mutation c.889G>A (p.C162Y) in *COCH* were followed up to observe the disease course of DFNA9. Based on the mutation sites in cochlin and their correlation with molecular defects in cochlin, we made the assumptions that vestibular dysfunction may be caused by disruption of normal cleavage to produce secreted LCCL domain fragments and that the cochlear symptoms may be closely related to aggregation of mutant cochlins. More DFNA9 families with different genotypes and phenotypes are needed to confirm the assumptions.

Although the variation of genotype-phenotype correlation in DFNA9 is only limited to cochlear and vestibular dysfunctions, this is of clinical importance for differential diagnosis. The affected members in family #32 had the phenotype of deafness without any vestibular symptoms, quite different from the phenotype in family #208. Genetic background or environmental factors may be the factors that determine the presence of disrupted cleavage of secreted LCCL domain fragments or aggregation of the p.C162Y mutant cochlin. For example, the severe vertigo attacks and sudden deafness in family #208 always appeared in situations of feeling tired or working for a long period.

Some DFNA9 cases with cochlin mutations in the LCCL domain had a phenotype similar to Ménière’s disease [27, 28]. The proband in family #208 also presented with recurrent attacks of combined vestibular and auditory symptoms, and this is the first study to follow up vestibular and cochlear functions at acute attack and interval periods for more than 3 years in DFNA9 due to cochlin mutations. The hearing symptoms are different between DFNA9 and Ménière’s disease. Unlike the fluctuation of sensorineural hearing loss starting from low frequencies in Ménière’s disease, DFNA9 patients show progressive deterioration of sensorineural hearing loss starting from high frequencies then slowly progressing to all frequencies, probably due to the different pathogeneses of these two diseases.

ECochG is an electrophysiological test that can reflect elevation of inner ear pressure and distention of the basilar membrane. ECochG measures the ratio of summating potential to nerve action potential (SP/AP) in response to auditory stimuli. A SP/AP ratio greater than 37% is thought to be a sign of endolymphatic hydrops seen in Ménière’s disease [29, 30]. The ECochG results for the proband in family #208 showed a continued and slowly progressive summating potential. The ECochG results together with the OAE results are the signs of hair cell dysfunction. To the best of our knowledge, ECochG has not been used to test DFNA9 patients previously, and the consistently abnormal results in this family suggest that it is a valuable tool for the diagnosis of DFNA9.

The otolithic input to the sternocleidomastoid principally arises from the saccule [31, 32], and the cVEMP test can reflect saccular function. Cochlin has been reported to be abundantly expressed in saccule and utricle. The mutant cochlin accumulated in these sites may influence saccular function, consistent with the test results for the proband in family #208. Interestingly, VEMP tests were abnormal in all DFNA9 patients tested to date [14, 15], which may suggest that VEMP tests are sensitive indicators for DFNA9. In addition, our recuperative caloric testing and head impulse testing results indicate that semicircular canal functions may be normal in the intervals of the attacks.

## Supporting Information

S1 Table Targeted genes for targeted next-generation sequencing. (PDF)

S2 Table Seven variants identified by targeted next-generation exome sequencing. (PDF)

## References

[1] Heller S, Sheane CA, Javed Z, Hudspeth AJ. Molecular markers for cell types of the inner ear and candidate genes for hearing disorders. Proc Natl Acad Sci USA. 1998; 95: 11400–11405. 9736748 10.1073/pnas.95.19.11400 PMC21654

[2] Robertson NG, Resendes BL, Lin JS, Lee C, Aster JC, Adam JC, et al Inner ear localization of mRNA and protein products of COCH, mutated in the sensorineural deafness and vestibular disorder, DFNA9. Hum Mol Genet. 2001; 10: 2493–2500. 11709536 10.1093/hmg/10.22.2493

[3] Py BF, Gonzalez SF, Long K, Kim MS, Kim YA, Zhu H, et al Cochlin produced by follicular dendritic cells promotes antibacterial innate immunity. Immunity. 2013; 38: 1063–1072. 10.1016/j.immuni.2013.01.015 23684986 PMC3758559

[4] Robertson NG, Lu L, Heller S, Merchant SN, Eavey RD, McKenna M, et al Mutations in a novel cochlear gene cause DFNA9, a human nonsyndromic deafness with vestibular dysfunction. Nat Genet. 1998; 20: 299–303. 10.1038/3118 9806553

[5] de Kok YJ, Bom SJ, Brunt TM, Kemperman MH, van Beusekom E, van der Velde-Visser SD, et al A Pro51Ser mutation in the COCH gene is associated with late onset autosomal dominant progressive sensorineural hearing loss with vestibular defects. Hum Mol Genet. 1999; 8: 361–366. 9931344 10.1093/hmg/8.2.361

[6] Kamarinos M, McGill J, Lynch M, Dahl H. Identification of a novel COCH mutation, I109N, highlights the similar clinical features observed in DFNA9 families. Hum Mutat. 2001; 17: 351 10.1002/humu.37 11295836

[7] Nagy I, Horvath M, Trexler M, Repassy G, Patthy L. A novel COCH mutation, V104del, impairs folding of the LCCL domain of cochlin and causes progressive hearing loss. J Med Genet. 2004; 41: e9 10.1136/jmg.2003.012286 14729849 PMC1757273

[8] Collin RW, Pauw RJ, Schoots J, Huygen PL, Hoefsloot LH, Cremers CW, et al Identification of a novel COCH mutation, G87W, causing autosomal dominant hearing impairment (DFNA9). Am J Med Genet A. 2006; 140: 1791–1794. 10.1002/ajmg.a.31354 16835921

[9] Baek JI, Cho HJ, Choi SJ, Kim LS, Zhao C, Sagong BR, et al The Trp117Arg mutation of the COCH gene causes deafness in Koreans. Clin Genet. 2010; 77: 399–403. 10.1111/j.1399-0004.2009.01362.x 20447147

[10] Hildebrand MS, Gandolfo L, Shearer AE, Webster JA, Jensen M, Kimberling WJ, et al A novel mutation in COCH—implications for genotype-phenotype correlations in DFNA9 hearing loss. Laryngoscope. 2010; 120: 2489–2493. 10.1002/lary.21159 21046548 PMC3329724

[11] Gallant E, Francey L, Fetting H, Kaur M, Hakonarson H, Clark D, et al Novel COCH mutation in a family with autosomal dominant late onset sensorineural hearing impairment and tinnitus. Am J Otolaryngol. 2013; 34: 230–235. 10.1016/j.amjoto.2012.11.002 23374487

[12] Chen DY, Chai YC, Yang T, Wu H. Clinical characterization of a novel COCH mutation G87V in a Chinese DFNA9 family. Int J Pediatr Otorhinolaryngol. 2013; 77: 1711–1715. 10.1016/j.ijporl.2013.07.031 23993205

[13] Jung J, Kim HS, Lee MG, Yang EJ, Choi JY. Novel COCH p.V123E mutation, causative of DFNA9 sensorineural hearing loss and vestibular disorder, shows impaired cochlin post-translational cleavage and secretion. Hum Mutat. 2015; 36: 1168–1175. 10.1002/humu.22855 26256111

[14] Street VA, Kallman JC, Robertson NG, Kuo SF, Morton CC, Phillips JO. A novel DFNA9 mutation in the vWFA2 domain of COCH alters a conserved cysteine residue and intrachain disulfide bond formation resulting in progressive hearing loss and site-specific vestibular and central oculomotor dysfunction. Am J Med Genet A. 2005; 139A: 86–95. 10.1002/ajmg.a.30980 16261627

[15] Yuan HJ, Han DY, Sun Q, Yan D, Sun HJ, Tao R, et al Novel mutations in the vWFA2 domain of COCH in two Chinese DFNA9 families. Clin Genet. 2008; 73: 391–394. 10.1111/j.1399-0004.2008.00972.x 18312449

[16] Cho HJ, Park HJ, Trexler M, Venselaar H, Lee KY, Robertson NG, et al A novel COCH mutation associated with autosomal dominant nonsyndromic hearing loss disrupts the structural stability of the vWFA2 domain. J Mol Med. 2012; 90: 1321–1331. 10.1007/s00109-012-0911-2 22610276 PMC4361775

[17] Gao J, Xue J, Chen L, Ke X, Qi Y, Liu Y. Whole exome sequencing identifies a novel DFNA9 mutation, C162Y. Clin Genet. 2013; 83: 477–481. 10.1111/cge.12006 22931125

[18] Robertson NG, Skvorak AB, Yin Y, Weremowicz S, Johnson KR, Kovatch KA, et al Mapping and characterization of a novel cochlear gene in human and in mouse: a positional candidate gene for a deafness disorder, DFNA9. Genomics. 1997; 46: 345–354. 10.1006/geno.1997.5067 9441737

[19] Bom SJ, Kemperman MH, Huygen PL, Luijendijk MW, Cremers CW. Cross-sectional analysis of hearing threshold in relation to age in a large family with cochleovestibular impairment thoroughly genotyped for DFNA9/COCH. Ann Otol Rhinol Laryngol. 2003; 112: 280–286. 12656423 10.1177/000348940311200316

[20] Bae SH, Robertson NG, Cho HJ, Morton CC, Jung DJ, Baek JI, et al Identification of pathogenic mechanisms of COCH mutations, abolished cochlin secretion, and intracellular aggregate formation: genotype-phenotype correlations in DFNA9 deafness and vestibular disorder. Hum Mutat. 2014; 35: 1506–1513. 10.1002/humu.22701 25230692 PMC4373469

[21] Pauw RJ, Huygen PL, Colditz GM, Cremers CW. Phenotype analysis of an Australian DFNA9 family with the 1109N COCH mutation. Ann Otol Rhinol Laryngol. 2011; 120: 414–421. 21774451 10.1177/000348941112000612

[22] Liepinsh E, Trexler M, Kaikkonen A, Weigelt J, Banyai L, Patthy L, et al NMR structure of the LCCL domain and implications for DFNA9 deafness disorder. EMBO J. 2001; 20: 5347–5353. 10.1093/emboj/20.19.5347 11574466 PMC125649

[23] Stelma F, Bhutta MF. Non-syndromic hereditary sensorineural hearing loss: review of the genes involved. J Laryngol Otol. 2014; 128: 13–21. 10.1017/S0022215113003265 24423691

[24] Pauw RJ, Huygen PL, Collin RW, Cruysberg JR, Hoefsloot LH, Kremer H, et al Phenotype description of a novel DFNA9/COCH mutation, I109T. Ann Otol Rhinol Laryngol. 2007; 116: 349–357. 17561763 10.1177/000348940711600506

[25] Usami S, Takahashi K, Yuge I, Ohtsuka A, Namba A, Abe S, et al Mutations in the COCH gene are a frequent cause of autosomal dominant progressive cochleo-vestibular dysfunction, but not of Meniere's disease. Eur J Hum Genet. 2003; 11: 744–748. 10.1038/sj.ejhg.5201043 14512963

[26] Sun Y, Zhang Z, Cheng J, Lu Y, Yang CL, Luo YY, et al A novel mutation of EYA4 in a large Chinese family with autosomal dominant middle-frequency sensorineural hearing loss by targeted exome sequencing. J Hum Genet. 2015; 60: 299–304. 10.1038/jhg.2015.19 25809937

[27] Fransen E, Verstreken M, Verhagen WI, Wuyts FL, Huygen PL, D'Haese P, et al High prevalence of symptoms of Meniere's disease in three families with a mutation in the COCH gene. Hum Mol Genet. 1999; 8: 1425–1429. 10400989 10.1093/hmg/8.8.1425

[28] Harcourt J, Barraclough K, Bronstein AM. Meniere's disease. BMJ. 2014; 349: g6544 10.1136/bmj.g6544 25391837

[29] Lamounier P, Gobbo DA, Souza TS, Oliveira CA, Bahmad F Jr. Electrocochleography for Meniere's disease: is it reliable? Braz J Otorhinolaryngol. 2014; 80: 527–532. 10.1016/j.bjorl.2014.08.010 25443316 PMC9442722

[30] Sajjadi H, Paparella MM. Meniere's disease. Lancet. 2008; 372: 406–414. 10.1016/S0140-6736(08)61161-7 18675691

[31] Deriu F, Ortu E, Capobianco S, Giaconi E, Melis F, Aiello E, et al Origin of sound-evoked EMG responses in human masseter muscles. J Physiol. 2007; 580: 195–209. 10.1113/jphysiol.2006.123240 17234698 PMC2075422

[32] Ikezono T, Shindo S, Ishizaki M, Li L, Tomiyama S, Takumida M, et al Expression of cochlin in the vestibular organ of rats. J Otorhinolaryngol Relat Spec. 2005; 67: 252–258. 10.1159/000089404 16374056

