# Supplementary material for: Different Phenotypes of the Two Chinese Probands with the Same c.889G>A (p.C162Y) Mutation in COCH Gene Verify Different Mechanisms Underlying Autosomal Dominant Nonsyndromic Deafness 9
Source: PLoS One. 2017 Jan 18;12(1):e0170011. doi: 10.1371/journal.pone.0170011 (PMC5243127; doi:10.1371/journal.pone.0170011)
Supplement: S1 Table — (PDF) [file pone.0170011.s001.pdf]

S1 Table. The targeted genes for targeted next-generation sequencing.

ACTG1	ALX3	BSND	CABP2	CCDC50	CDH23	CEACAM16
CHD7	CIB2	CLDN14	CLPP	CLRN1	COCH	COL11A1
COL11A2	COL2A1	COL4A3	COL4A4	COL4A5	COL4A6	COL9A1
COL9A2	COMT2	CRYM	DFNA5	DFNB31	DFNB59	DIABLO
DIAPH1	DIAPH3	DSPP	ECM1	EDN3	EDNRB	ELMOD3
ESPN	ESRRB	EYA1	EYA4	FGF3	FGF8	FGFR1
FGFR3	FLNA	FOXI1	FREM1	GATA3	GIPC3	GJB2
GJB3	GJB6	GPR98	GPSM2	GRHL2	GRXCR1	HARS
HARS2	HGF	HMX1	HOXA2	HSD17B4	IL13	ILDR1
KARS	KCNE1	KCNJ10	KCNQ1	KCNQ4	KRT9	LAMA3
LARS2	LHFPL5	LOXHD1	LRTOMT	MARVELD2	MIR96	MITF
MSRB3	MYH14	MYH9	MYO15A	MYO1A	MYO3A	MYO6
MYO7A	NDP	NF2	OTOA	OTOF	P2RX2	PABPN1
PAX3	PCDH15	PDZD7	PNPT1	POLR1C	POLR1D	POU3F4
POU4F3	PROK2	PROKR2	PRPS1	PTPRQ	RDX	RPGR
SALL1	SALL4	SEC23A	SEMA3E	SERPINB6	SIX1	SIX5
SLC17A8	SLC26A4	SLC26A5	SMPX	SNAI2	SOX10	STRC
TBC1D24	TCOF1	TECTA	TIMM8A	TJP2	TMC1	TMIE
TMPRSS3	TNC	TPRN	TRIOBP	TRMU	TSPEAR	USH1C
USH1G	USH2A	WFS1				
