# Supplementary material for: Different Phenotypes of the Two Chinese Probands with the Same c.889G>A (p.C162Y) Mutation in COCH Gene Verify Different Mechanisms Underlying Autosomal Dominant Nonsyndromic Deafness 9
Source: PLoS One. 2017 Jan 18;12(1):e0170011. doi: 10.1371/journal.pone.0170011 (PMC5243127; doi:10.1371/journal.pone.0170011)
Supplement: S2 Table — (PDF) [file pone.0170011.s002.pdf]

S2 Table. Seven variants identified by targeted next-generation exome sequencing.

UniqID	MutPosition	Disease	Genetic Mode	SIFT	SIFT_predict	PolyPhen_2	PolyPhen_2_predict	MutationTaster	MutationTaster_predict
COCH-chr14-31349796	COCH:C162Y	DFNA9(autosomal dominant nonsyndromic sensorineural deafness 9)	AD	0	Damaging	0.998	Probably_damaging	1	Disease_causing
FLNA-chrX-153582543	FLNA:R1837C	Intestinal pseudoobstruction	XR	0	Damaging	0.998	Probably_damaging	1	Disease_causing
KCNJ10-chr1-160011511	KCNJ10:R271H	Deafness, Autosomal Recessive 4, With Enlarged Vestibular Aqueduct.	AR	0.03	Damaging	0.11	Benign	1	Disease_causing
KCNQ1-chr11-2610034	KCNQ1:P448R	Long Qt Syndrome 1 and Jervell And Lange-Nielsen Syndrome.	AD	0.55	Tolerable	0.051	Benign	1	Polymorphism
LOXHD1-chr18-44137388	LOXHD1:D1094G	Deafness, Autosomal Recessive 77	AR	0.03	Damaging	1	Probably_damaging	1	Disease_causing
RPGR-chrX-38164037	RPGR:A262G	Retinitis Pigmentosa, X-Linked, And Sinorespiratory Infections, With Or Without Deafness	XD	1	Tolerable	0.182	Benign	1	Polymorphism
SIX1-chr14-61113151	SIX1:Q235H	Deafness, Autosomal Dominant 23	AD	0.32	Tolerable	0.947	Possibly_damaging	1	Disease_causing
